# A short-term treatment with tumor necrosis factor-alpha enhances stem cell phenotype of human dental pulp cells

**DOI:** 10.1186/scrt420

**Published:** 2014-02-28

**Authors:** Mayu Ueda, Takuo Fujisawa, Mitsuaki Ono, Emilio Satoshi Hara, Hai Thanh Pham, Ryu Nakajima, Wataru Sonoyama, Takuo Kuboki

**Affiliations:** 1 Department of Oral Rehabilitation and Regenerative Medicine, Okayama University Graduate School of Medicine, Dentistry and Pharmaceutical Sciences, 2-5-1, Shikata-cho, Okayama 700-8525, Japan

## Abstract

**Introduction:**

During normal pulp tissue healing, inflammatory cytokines, such as tumor necrosis factor-alpha (TNF-α) or interleukins, act in the initial 48 hours (inflammatory phase) and play important roles not only as chemo-attractants of inflammatory cells and stem/progenitor cells but also in inducing a cascade of reactions toward tissue regeneration or reparative dentin formation or both. Previous reports have shown that inflammatory cytokines regulate the differentiation capacity of dental pulp stem/progenitor cells (DPCs), but none has interrogated the impact of these cytokines on the stem cell phenotype of stem/progenitor cells. This study investigated the effects of a short-term treatment with TNF-α on the stem cell phenotype and differentiation ability of human DPCs.

**Methods:**

An *in vivo* mouse model of pulp exposure was performed for analysis of expression of the mesenchymal stem cell marker CD146 in DPCs during the initial stage of inflammatory response. For *in vitro* studies, human DPCs were isolated and incubated with TNF-α for 2 days and passaged to eliminate TNF-α completely. Analysis of stem cell phenotype was performed by quantification of cells positive for mesenchymal stem cell markers SSEA-4 (stage-specific embryonic antigen 4) and CD146 by flow cytometry as well as by quantitative analysis of telomerase activity and mRNA levels of *OCT-4* and *NANOG*. Cell migration, colony-forming ability, and differentiation toward odontogenesis and adipogenesis were also investigated.

**Results:**

The pulp exposure model revealed a strong staining for CD146 during the initial inflammatory response, at 2 days after pulp exposure. *In vitro* experiments demonstrated that a short-term (2-day) treatment of TNF-α increased by twofold the percentage of SSEA-4^+^ cells. Accordingly, STRO-1, CD146, and SSEA-4 protein levels as well as *OCT-4* and *NANOG* mRNA levels were also significantly upregulated upon TNF-α treatment. A short-term TNF-α treatment also enhanced DPC function, including the ability to form cell colonies, to migrate, and to differentiate into odontogenic and adipogenic lineages.

**Conclusions:**

A short-term treatment with TNF-α enhanced the stem cell phenotype, migration, and differentiation ability of DPCs.

## Introduction

The normal healing process of an injured tissue is characterized by very orderly and distinct but overlapping phases: hemostasis, inflammation, proliferation, and remodeling [1]. During the inflammation phase, chemokines and pro-inflammatory cytokines such as interleukins (for example, IL-1α, IL-1β, and IL-6) and tumor necrosis factor-alpha (TNF-α) are released by activated macrophages at the injured site and initiate the inflammatory cascade. The inflammatory period has its peak in the initial 48 hours and is important not only for recruiting leucocytes but also for activation of surrounding connective tissue cells, including stem/progenitor cells, that migrate to the injured site and contribute to tissue healing, which characterizes the proliferative and remodeling phases [2,3]. On the other hand, feedback signaling from the cells surrounding the injury site modulates the activation of resident macrophages by secretion of anti-inflammatory factors such as TNF-α-stimulated gene/protein 6 (TSG-6), prostaglandin E_2_ (PGE_2_), and interleukin-1 receptor antagonist (IL-1ra) to eventually suppress or terminate the inflammatory phase.

Such a synchronized and feedback-controlled regulation of inflammation and regeneration phases is crucial for normal tissue healing, and alteration in inflammatory signals has been reported to disrupt the normal tissue healing. For instance, gene deletion of core inflammatory cytokines, such as TNF-α, has been associated with impaired healing or pathogenic tissue response in mice [4,5]. On the other hand, extended duration of the inflammatory phase, such as in the case of chronic inflammation, is widely known to repress complete tissue regeneration.

Dental pulp is frequently submitted to damage or injury, and, in most cases, dental pulp cells (DPCs) deposit reparative or tertiary dentin in response to the injury [6]. In this context, previous *in vitro* studies have shown that a short-term treatment with TNF-α or IL-1β (or both) induces matrix deposition and increases the expression of odontogenic marker genes dentinsialoprotein (*DSP*), dentin matrix protein (*DMP-1*), and osteocalcin (*OCN*) in DPCs [7,8]. On the other hand, a long-term stimulation with TNF-α suppresses the differentiation ability of DPCs [8,9].

Therefore, it is clear that pro-inflammatory cytokines modulate the function and differentiation ability of mesenchymal stem/progenitor cells. However, no study has investigated the effect of inflammatory cytokines on the stem cell phenotype of DPCs so far. In this study, we show, for the first time, an increase in CD146-positive cells during the initial inflammatory phase in an *in vivo* pulp exposure model. Additionally, subsequent *in vitro* experiments revealed that a short-term stimulation with TNF-α, but not with IL-1β or IL-6, enhanced the stem cell phenotype of human DPCs as determined by telomerase activity, analysis of gene expression levels of *OCT-4* and *NANOG*, and expression of the mesenchymal stem cell surface markers SSEA-4 (stage-specific embryonic antigen 4) and CD146. Finally, we show that the increase in stem cell phenotype induced by TNF-α was associated with enhanced migration, colony formation, and multipotent differentiation ability of DPCs toward odontogenic and adipogenic lineages.

## Materials and Methods

### Pulp exposure model in mice

Six-week-old mice were anesthetized with intra-peritoneal injection of a mixture of xylazine (8 mg/kg; Bayer, Tokyo, Japan) and ketamine (80 mg/kg; Sankyo, Tokyo, Japan). Mandibular first molars were drilled with a diamond bur and air-turbine handpiece to expose the pulp, which was left untreated for 2 or 4 days. Subsequently, mice were sacrificed and mandibles were immediately immersed in embedding medium, frozen, and sectioned in 5 mm-thick sections in accordance with a previously described method [10]. The animals were treated in accordance with the Guidelines for Animal Research of Okayama University Dental School. The research protocol for animal experiments was approved by the Okayama University Ethics Committee (OKU-2013125).

### Human cell isolation and culture

Human DPCs were isolated from third molars or pre-molars of at least three adults (20 to 23 years old) at Okayama University Hospital, and written informed consent was obtained from all subjects in accordance with a previously reported method [11]. Guidelines and protocol for utilization of DPCs from human subjects were approved by the Okayama University Ethics Committee (#418). Cells were cultured with basal medium, consisting of alpha-minimum essential medium (α-MEM) (Gibco BRL, New York, NY, USA), 15% fetal bovine serum (FBS) (Invitrogen, Carlsbad, CA, USA), 100 mM L-ascorbic acid 2-phosphate (Wako Pure Chemical Industries, Osaka, Japan), 2 mM L-glutamine (Invitrogen), 100 units/mL penicillin (Sigma-Aldrich, St. Louis, MO, USA), and 100 mg/mL streptomycin (Sigma-Aldrich), at 37°C under 5% CO_2_ in air.

For pretreatment experiments with TNF-α, DPCs were cultured up to 60% confluence and further incubated with 10 ng/mL of recombinant human TNF-α (rhTNF-α) (R&D Systems, Minneapolis, MN, USA) for 2 days. After reaching sub-confluency, cells were passaged once with accutase (Innovative Cell Technologies Inc., San Diego, CA, USA) to remove TNF-α completely before assays. DPCs up to the eighth passage were used in this study.

Neutralization of TNF-α receptor 1 (TNFR1) was performed with human anti-TNFRI antibody (TNFR1_AB_; MAB625, R&D). IL-1 and IL-6 were purchased from R&D Systems.

### Cell viability

Cell viability was assessed by a colorimetric assay based on the bioreduction of a tetrazolium compound—3-(4,5-dimethylthiazol-2-yl)-5-(3-carboxymethoxyphenyl)-2-(4-sulfophenyl)-2H-tetrazolium, inner salt; MTS—by viable cells (CellTiter 96 AQueous One Solution Cell Proliferation Assay; Promega, Madison, WI, USA) in accordance with the instructions of the manufacturer.

### Multipotent differentiation

DPCs were induced to differentiate toward the odontogenic lineage, based on a previous reported protocol with a few modifications [12,13]. Briefly, DPCs were cultured up to 80% confluence and thereafter incubated in mineralization medium—basal medium containing 10 nM dexamethasone (Sigma-Aldrich), β-glycerophosphate (Sigma-Aldrich)—for 10 or 21 days. Cultures were analyzed for gene expression of alkaline phosphatase (*ALP*), dentinsialophosphoprotein (*DSPP*), and osteopontin (*OPN*) or stained for calcified extracellular matrix by staining with 1% alizarin red S staining solution.

Induction of adipogenic differentiation of DPCs was performed under similar culture conditions of the odontogenic induction method, and cultures were incubated in basal medium containing 1 μg/mL insulin (Sigma-Aldrich), 0.5 mM 1-methyl-3-isobutylxanthine (Sigma-Aldrich), and 60 μM indomethacin (Sigma-Aldrich) for 14 or 30 days. After induction, cultures were analyzed for gene expression of adipocyte protein 2 (*AP2*) or stained for oil drops with 0.5% Oil Red O staining solution.

### Reverse transcription and real-time polymerase chain reaction

Total RNA from DPC cultures was extracted with RNeasy (Qiagen, Hilden, Germany) and purified by removing genomic DNA with RNase-Free DNase set (Qiagen), as described previously [14]. Expression levels of the target genes were normalized to that of the reference gene *S29*. Primer sequences were the following: *S29* (BC032813) sense: 5′-ACACTGGCGCACATATTGAGG-3′, *S29* anti-sense: 5′- TCTCGCTCTTGTCGTGTCTGTTC-3′; *OCT-4* (NM_001159542) sense: 5′-CCGAGTGTGGTTCTGTAAC-3′, *OCT-4* anti-sense: 5′-GAAAGGGACCGAGGAGTA-3′; *NANOG* (NM_024865) sense: 5′-TCTCCAACATCCTGAACCT-3′, *NANOG* anti-sense: 5′-GCGTCACACCATTGCTAT-3′; *ALP* (NM_000478) sense: 5′-GCACCGCCACCGCCTACC-3′; *ALP* anti-sense: 5′-CCACAGATTTCCCAGCGTCCTTG-3′; *DSPP* (NM_014208) sense: 5′-TGGAGCCACAAACAGAAGCAACAC-3′; *DSPP* anti-sense: 5′-TGGACAACAGCGACATCCTCATTG-3′; *OPN* (BC007016) sense: 5′-ATGTGATTGATAGTCAGGAACTT-3′; *OPN* anti-sense: 5′-GTCTACAACCAGCATATCTTCA-3′; *OCN* (NM_199173) sense: 5′-CAGAGTCCAGCAAAGGTG-3′; *OCN* anti-sense: 5′-AGCCATTGATACAGGTAGC-3′; and *AP2* (BC104842) sense: 5′-GGGTGGGCAGACTGTGGACTC-3′, *AP2* anti-sense: 5′-AGGGAGCAGAAGAGAAGTGTCAGG-3′.

### Flow cytometry

DPCs were dissociated with accutase and filtered through a 70-μm cell strainer, washed, resuspended in phosphate-buffered saline (PBS) containing 1% FBS, and incubated with antibodies (anti-human SSEA-4, CD29, CD34, CD44, CD45, CD90, and CD146 antibody; BD Biosciences, San Jose, CA, USA) for 30 minutes on ice [15]. Cells were washed and subjected to flow cytometry (FCM) analysis by MACSQuant Analyzer (Miltenyi Biotec, Bergisch Gladbach, Germany) or Accuri™ C6 (BD Biosciences).

### Immunofluorescence staining

Immunostaining of cryosections was performed by initial blocking with 10% normal goat serum, followed by incubation with anti-CD-146 antibody (Abcam, Cambridge, MA, USA) or IgG (Abcam), wash, and incubation with second antibody AlexaFluor 488 conjugated IgG (Invitrogen).

DPCs were seeded onto 96-well plates and cultured for 24 hours in basal medium. The cells were subsequently fixed in 4% paraformaldehyde (PFA) for 15 minutes, permeabilized with PBS containing 0.25% Triton X-100 for 10 minutes, blocked with 10% normal goat serum, and incubated with primary antibodies anti-STRO-1 (Developmental Studies Hybridoma Bank, Iowa City, IA, USA), anti-SSEA-4 (Millipore, Billerica, MA, USA), or anti-CD146 (Abcam) for 1 hour or overnight. In negative controls, primary antibodies were replaced by an appropriate isotype-matched negative IgM or IgG. Cells were incubated with AlexaFluor 488 conjugated anti-mouse IgM (Invitrogen) or IgG (Invitrogen).

Cell nuclei were stained with 4′6-diamidino-2-phenylindole (DAPI) (Invitrogen). Analysis of immunofluorescence staining was performed under a microscope (Biozero BZ-8000; Keyence Corp., Osaka, Japan).

### Colony-forming assay

Colony-forming unit-fibroblast (CFU-F) assay was performed by seeding 100 cells in a 25-cm^2^ culture flask and culture for 2 weeks. Cells were washed with PBS and stained with 0.1% toluidine blue contained in 1% PFA overnight. On the following day, flasks were washed to remove excess dye, and only stained clusters containing more than 50 cells were counted as colonies.

### Migration assay

Migration assay was performed in accordance with the Boyden chamber method, using cell culture inserts with a light-opaque polyethylene terephthalate 8-μm microporous membrane (BD Falcon HTS FluoroBlok inserts; BD Biosciences). DPCs were dissociated with accutase, counted, and incubated with 5 μg/mL of calcein AM (BD Biosciences) for 15 minutes at 37°C and 5% CO_2_. Cells were washed with PBS, centrifuged, and resuspended with serum-starved (1% FBS) medium. Cells were counted one more time, seeded in the upper chamber (cell insert), and incubated for 24 hours. Subsequently, cells were fixed with 4% PFA, washed with PBS, and observed under the microscope. The total number of migrated cells observed in the bottom of the chamber was counted in four different pictures taken per chamber/insert.

### Telomerase activity

Telomerase activity in DPCs was detected by using a quantitative telomerase detection kit (Allied Biotech, Inc., Ijamsville, MD, USA) in accordance with the protocol of the manufacturer.

### Statistical analysis

An unpaired Student *t* test or one-way analysis of variance followed by Tukey *post hoc* correction test was used for statistical analysis with GraphPad Prism (GraphPad Software Inc., San Diego, CA, USA). *P* values of less than 0.05 were considered to be statistically significant.

## Results

### Increase in CD146^+^ cells during pulpal inflammatory response *in vivo*

**Figure 1:**
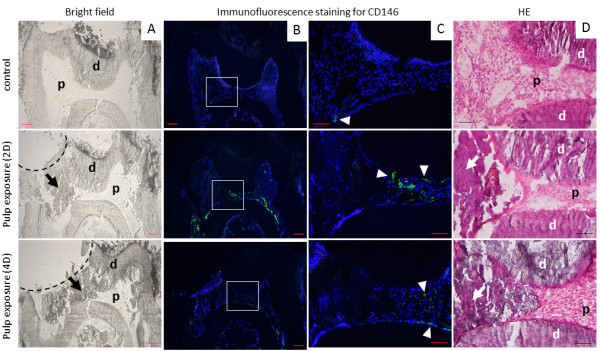
**Pulp exposure model in mice. (A)** Bright-field image of cryosections of mouse lower first molar: control and pulp exposure model at 2 and 4 days after injury. **(B,C)** Immunofluorescence staining for CD146. Note a strong signal intensity corresponding to CD146 at 2 days after pulp exposure. **(D)** Hematoxylin and eosin staining of sections. Dotted lines indicate the drilled dentin with clear pulp exposure, arrows show cellular debris, and squares indicate the area shown at high magnification in **(C)**. Arrowheads indicate CD146^+^ cells. d, dentin; p, dental pulp. Scale bars = 100 μm **(A,B)** and 50 μm **(C,D)**. Images are representative of three independent samples.

In an attempt to identify a correlation between the inflammatory response and changes in stem cell markers of DPCs *in vivo*, we performed immunostaining for CD146^+^ cells after 2 and 4 days of pulp exposure. Interestingly, there was a marked increase in the number of CD146^+^ cells at 2 days, and this subsequently decreased at day 4 after pulp exposure (Figure 1). These results indicate that an inflammatory response can induce remarkable increases in CD146^+^ cells.

### Effect of inflammatory cytokines on stem cell phenotype of human dental pulp cells *in vitro*

**Figure 2:**
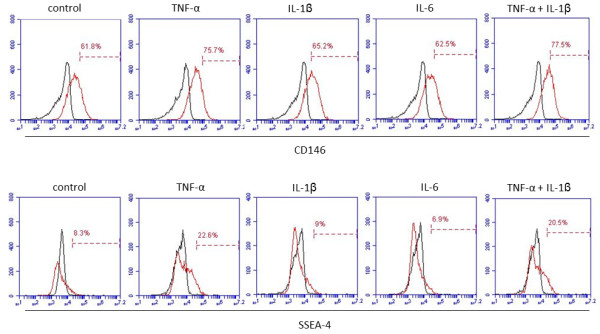
**Comparison of the effect of key inflammatory regulators on the stem cell phenotype of dental pulp stems (DPCs).** Tumor necrosis factor-alpha (TNF-α) was the only cytokine that remarkably increased the percentage of cells positive for CD146 and stage-specific embryonic antigen 4 (SSEA-4). Although TNF-α and interleukin-1-beta (IL-1β) have been reported to present a synergistic effect, the combination of them did not enhance the TNF-α effect. Results are representative of at least three independent experiments.

To address whether inflammatory cytokines could regulate the stem cell phenotype of DPCs, we proceeded to further *in vitro* experiments to analyze the effect of key regulators of inflammatory response—TNF-α, IL-1β, and IL-6—on the stem cell phenotype of human DPCs, which were previously characterized by surface antigen expression before assays (Additional file 1: Figure S1). As shown in Figure 2, TNF-α remarkably increased the number of cells positive for CD146 and SSEA-4, whereas no significant effect could be observed by IL-1β or IL-6 stimulation. Additionally, since TNF-α and IL-1β are known to have synergistic effects, a combination of the two cytokines was also examined; however, no substantial alteration in either CD146 or SSEA-4 markers was observed between TNF-α alone or in combination with IL-1β (Figure 2).

### Optimization of the experimental protocol with tumor necrosis factor-alpha

Next, we investigated the effect of TNF-α on the stemness of DPCs. To optimize the experimental protocol, we first analyzed DPC viability and changes in stem cell markers in response to increasing doses or increasing incubation periods with TNF-α. As shown in Additional file 2: Figure S2A, TNF-α did not affect the viability of DPCs at any concentration. The TNF-α-induced increase in the number of SSEA-4^+^ cells was also not affected by different dosages of TNF-α (Additional file 2: Figure S2B). Therefore, we applied TNF-α at a concentration of 10 ng/mL in the subsequent assays.

On the other hand, a time-dependent analysis of the TNF-α effect showed a significant decrease in cell viability when the incubation period extended for consecutive 6 days (Additional file 2: Figure S2C), and this could explain, in part, the inhibitory effect of long-term treatment of TNF-α on DPC differentiation (Additional file 2: Figure S2D, right panels). We also incubated DPCs with TNF-α for consecutive 4 days and observed no changes in CD146 or *OCT-4* mRNA levels (Additional file 2: Figure S2E-F). Therefore, based on the *in vivo* results showing an intense staining for CD146 at 2 days after pulp exposure as well as on the fact that the inflammatory period is normally terminated within 48 hours, we established the experimental protocol by incubating DPCs with TNF-α for 2 days, then passaged cells in order to completely eliminate TNF-α from medium, and cultured the cells for additional days before utilization in subsequent assays.

### A short-term tumor necrosis factor-alpha treatment enhances stem cell phenotype, function, and differentiation of dental pulp stems

**Figure 3:**
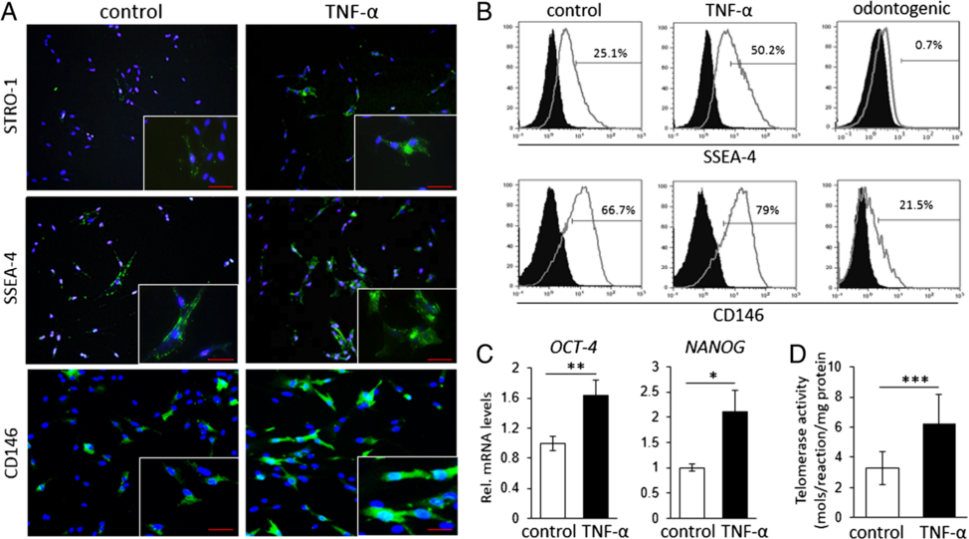
**A short-term treatment with tumor necrosis factor-alpha (TNF-α) enhances the stem cell phenotype of dental pulp stems (DPCs). (A)** Immunofluorescence analysis of expression of stem cell markers STRO-1, CD146, and stage-specific embryonic antigen 4 (SSEA-4) upon TNF-α treatment. Treatment with TNF-α markedly increased the number of cells positive for the three markers. **(B)** Flow cytometric analysis of expression of SSEA-4 and CD146. TNF-α treatment increased by twofold the number of cells positive for SSEA-4. Odontogenic differentiation decreased the percentage of SSEA-4^+^ and CD146^+^ cells, demonstrating that expression of these markers is closely related to their differentiation stage. **(C)** Effects of TNF-α pretreatment on mRNA levels of stem cell markers *OCT-4* and *NANOG*. TNF-α treatment significantly increased the gene expression of *OCT-4* and *NANOG*. **(D)** Analysis of telomerase activity between TNF-α-treated and untreated DPCs. TNF-α treatment significantly increased telomerase activity of DPCs. *P <0.05, **P <0.01, unpaired *t* test. Images and graphs are representative of at least three different experiments.

Immunofluorescence studies showed that a few DPCs were stained positively for STRO-1, CD146, and SSEA-4 (Figure 3A, left panels). Interestingly, treatment with TNF-α markedly enhanced the percentage of cells that stained positive for the three markers (Figure 3A, right panels). Consistently, FCM analysis also demonstrated an increase in the percentage of cells positive for SSEA-4 and CD146 upon TNF-α treatment (Figure 3B, left and middle panels), with a notable decrease after odontogenic differentiation (Figure 3B, right panels), indicating that expression of these cell surface markers is associated directly with their differentiation stage. Consistently, TNF-α also increased mRNA levels of *OCT-4* and *NANOG* as well as telomerase activity of DPCs (Figure 3C,D).

**Figure 4:**
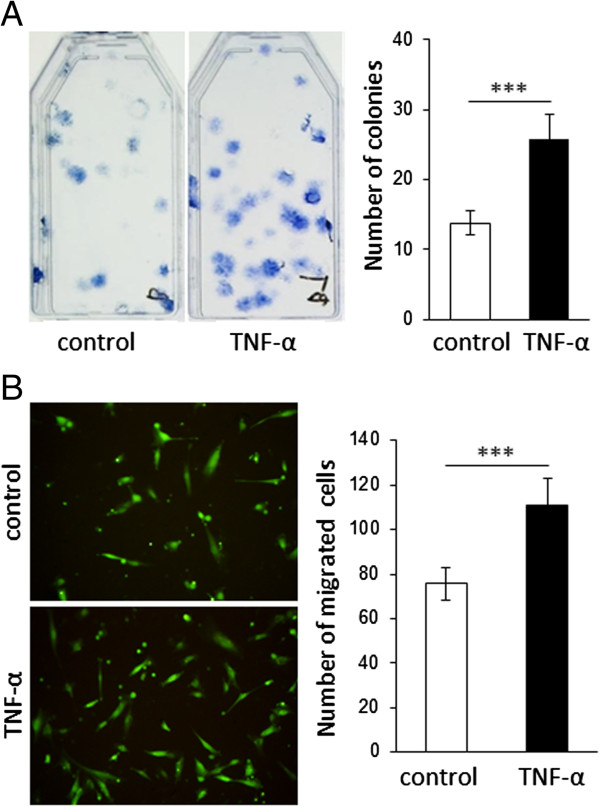
**A short-term treatment with tumor necrosis factor-alpha (TNF-α) enhances migration and colony-forming ability of dental pulp stems (DPCs). (A)** Analysis of colony-forming ability of DPCs. Treatment with TNF-α increased the ability of DPCs to form colonies. The graph on the right shows the quantitative analysis of the total number of cell clusters containing more than 50 cells. Data are representative of three independent experiments performed with triplicate samples. **(B)** Migration assay of DPCs. The images on the left are representative of three different experiments. The graph on the right shows the quantitative analysis of the total number of migrated cells counted in four different pictures taken from each chamber. TNF-α-treated DPCs presented higher migration ability compared with untreated controls. ***P <0.001, unpaired *t* test.

To address the possibility that the increase in stem cell phenotype and telomerase activity could be associated with changes in the function of DPCs, we compared colony-forming ability, which is one major characteristic of mesenchymal stem/progenitor cells, as well as migration ability between TNF-α-treated and control DPCs. As shown in Figure 4, stimulation with TNF-α significantly enhanced the ability of DPCs to form colonies (Figure 4A) and to migrate (Figure 4B), as shown by either representative images or quantitative analysis of the assays.

**Figure 5:**
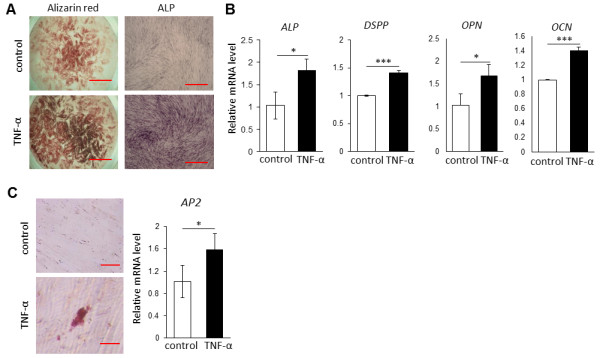
**Comparative analysis of differentiation capacity between tumor necrosis factor-alpha (TNF-α)-treated and untreated dental pulp stems (DPCs).** TNF-α treatment induced a great deposition of calcified matrix as detected by alizarin red staining and alkaline phosphatase **(A)** as well as the mRNA levels of *DSPP*, *ALP*, *OPN*, and *OCN***(B)**. Increased adipogenic differential ability was also observed in TNF-α-treated DPCs, determined by staining with oil red O and increase in mRNA levels of *AP2***(C)**. *P <0.05, ***P <0.001, unpaired *t* test. Data are representative of three independent experiments. Scale bar = 100 μm.

On the other hand, we also investigated the multipotent differentiation ability of DPCs, and the results showed that TNF-α treatment enhanced odontogenic and adipogenic differentiation of DPCs, as determined by ALP activity as well as alizarin red staining and oil red O staining, respectively (Figure 5A-C). Consistently, significant increases in the mRNA levels of odontogenic markers *ALP*, *DSPP*, *OPN*, and *OCN* (Figure 5B) and in the level of the adipogenic marker *AP2* were observed in the TNF-α-treated cells.

### TNFR1 blockade partially inhibits tumor necrosis factor-alpha effect

**Figure 6:**
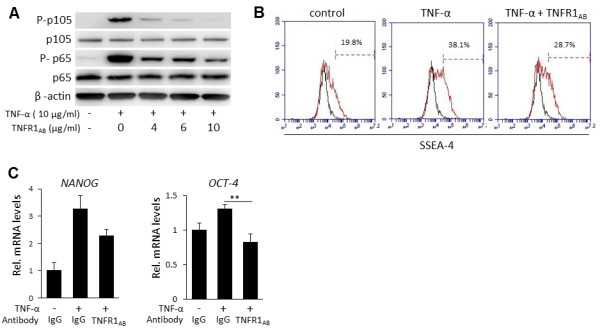
**Inhibition assays with antibody against tumor necrosis factor-receptor 1 (TNFR1**_**AB**_**). (A)** Analysis of nuclear factor-kappa-B (NF-κB) phosphorylation upon blockade of TNFR1. Dental pulp stems (DPCs) were incubated with different concentrations (0, 4, 6, and 10 μg/mL) of TNFR1_AB_ for 1 hour prior to treatment with TNF-α (10 ng/mL). Phosphorylation of NF-κB (p65, p105) was examined by Western blot at 5 minutes after stimulation. TNFR1_AB_ inhibited TNF-α-induced phosphorylation of p105 and p65 in a dose-dependent manner, and a maximum effect was observed with a concentration of 10 μg/mL. **(B)** Flow cytometric analysis of expression of stage-specific embryonic antigen 4 (SSEA-4). TNFR1_AB_ only partially inhibited TNF-α-induced increase in the number of SSEA-4^+^ cells. **(C)** Analysis of mRNA levels of *OCT-4* and *NANOG* upon inhibition of TNFR1. Although TNF-α treatment increased the gene expression of *OCT-4* and *NANOG*, TNFR1_AB_ nullified the TNF-α-induced increase *OCT-4* mRNA levels only. **P <0.01, one-way analysis of variance (ANOVA), Tukey test. Data are representative of at least three different experiments.

We performed numerous experiments in attempt to identify the mechanisms behind the TNF-α regulation of the stem cell phenotype of DPCs. Firstly, we performed inhibition assays by neutralization of TNFR1. To determine the optimal functional concentration of the TNFR1_AB_, we analyzed phosphorylation of nuclear factor-kappa-B (NF-κB) upon increasing doses of the antibody. As shown in Figure 6A, TNFR1_AB_ promoted a dose-dependent inhibition of TNF-α-induced NF-κB phosphorylation, and a satisfactory neutralization was achieved with a concentration of 10 μg/mL. We investigated changes in stem cell markers upon blockade of TNFR1. Nevertheless, as shown in Figure 6B, TNFR1_AB_ could only partially suppress the TNF-α-induced increase in the number of SSEA-4^+^ cells or in *NANOG* mRNA levels, although *OCT-4* mRNA levels could be completely nullified (Figure 6C).

Additionally, we examined a direct inhibition of NF-κB phosphorylation with chemical inhibitors (pyrrolidine dithiocarbamate and BAY 11–7082). The results, however, showed minimal changes in the stem cell phenotype of DPCs despite a complete inhibition of NF-κB phosphorylation (data not shown). These results demonstrate that the effect of TNF-α in enhancing DPC stem cell phenotype is in part through NF-κB signaling pathway, although a distinct mechanism is also involved in this process.

## Discussion

Inflammatory cytokines play fundamental roles during the process of wound healing and normal tissue regeneration. However, the mechanisms behind the cascade of biochemical events related to tissue inflammation/regeneration remain unclear. In the present work, we show that inflammation in the pulp is associated with increased expression of CD146, which could be related to stem/progenitor cell migration to the injured site or a possible enhancement of stem cell phenotype of stem/progenitor cells *in situ*. These results are in agreement with previous studies that also showed an increase in stem/progenitor cells during wound healing in a femur fracture model [3].

*In vitro* studies confirmed that TNF-α, when applied for a short period, induces significant and positive changes in the stem cell phenotype of DPCs by increasing not only the expression of stem cell markers but also telomerase activity of the cells. By FCM analysis, we demonstrated that stem cell phenotype (percentage of SSEA-4^+^ cells) [16] is intimately associated with their differentiation stage. Thus, we conjectured and further confirmed that TNF-α-enhanced stem cell phenotype resulted in increased differentiation ability of the cells.

The role of TNF-α on cell migration as a chemo-attractant factor has also been largely reported during tissue healing process, including migration of smooth muscle cells or mesenchymal stem cells [17-20]. Interestingly, however, our experimental model shows that TNF-α-enhanced cell migration is possibly associated with changes in the intracellular machinery that is extended even after subsequent cell passages, and this is a novel finding compared with the well-known chemo-attractant effect of TNF-α. Cell migration is known as a dynamic process regulated by actin filament remodeling, but only a few studies have reported on the effect of TNF-α on regulation of actin filament organization [21], and the mechanisms underlying this process are still largely unknown.

DPCs consist of a heterogeneous cell population; therefore, it is not clear from the present results whether the increase in the percentage of cells positive for stem cell surface markers is associated with an increase in the number of existing stem/progenitor cells or an increase in stem cell phenotype of differentiated cells. A recent article has demonstrated that NF-κB activity is required for cell reprogramming and induction of pluripotency by retroviral overexpression of reprogramming factors (Oct4, Sox2, Klf4, c-Myc) [22]. In the present investigation, since we analyzed the effect of TNF-α alone, we could not observe dramatic changes in stem cell phenotype as compared with reprogramming of induced pluripotent stem cells. Nonetheless, we demonstrate the importance of TNF-α and NF-κB signaling in regulating the stem cell phenotype of adult tissue-derived stem/progenitor cells.

Our findings also demonstrate that NF-κB is not the only pathway involved in the TNF-α-enhanced stem cell phenotype of DPCs, since neutralization of TNFR1 by TNFR1_AB_ or chemical blockade of NF-κB did not nullify the TNF-α-induced increase in stem cell markers, despite a complete inhibition of NF-κB phosphorylation. Additionally, we have investigated the involvement of other candidate signaling pathways, such as p38/MAP kinase; however, blockade of p38/MAPK with a specific inhibitor, SB-202190, could not rescue TNF-α-induced increase in the percentage of SSEA-4^+^ cells (data not shown). These results demonstrate that TNF-α regulation of stem cell phenotype involves complex mechanisms and intra-cellular pathways, and this demands additional comprehensive investigation.

## Conclusions

The present data show that short-term treatment with TNF-α significantly increases the stem cell phenotype of DPCs and their function and multipotent differentiation ability. These data are expected to greatly contribute to the understanding of the complex process involved in tissue healing/regeneration and to the research field of stem cell biology and regenerative medicine.

## Abbreviations

ALP: alkaline phosphatase; AP2: adipocyte protein 2; DPC: dental pulp cell; DSPP: dentinsialophosphoprotein; FBS: fetal bovine serum; FCM: flow cytometry; IL: interleukin; NF-κB: nuclear factor-kappa-B; OCN: osteocalcin; OCT-4/POU5F1: octamer-binding transcription factor 4/POU domain, class 5, transcription factor 1; OPN: osteopontin; PBS: phosphate-buffered saline; PFA: paraformaldehyde; SSEA-4: stage-specific embryonic antigen 4; TNF-α: tumor necrosis factor-alpha; TNFR1: tumor necrosis factor receptor 1; TNFR1AB: anti-tumor necrosis factor receptor 1 antibody.

## Competing interests

The authors declare that they have no competing interests.

## Authors’ contributions

MU performed most of the experiments and data analysis and drafted the manuscript. TF performed part of the experiments and data analysis and participated in the design of the study. MO participated in the design of the study and performed part of the experiments, data analysis, and manuscript writing. ESH performed part of the experiments and data analysis and wrote the manuscript. HTP performed inhibition assays. RN performed *in vivo* experiments, sectioning, and immunostaining studies. WS participated in the design of the study, data analysis, and manuscript preparation. TK participated in the design of the study, data analysis, and manuscript preparation. All authors read and approved the final manuscript.

## Supplementary Material

Additional file 1: Figure S1 Analysis of cell surface markers by flow cytometry. Isolated dental pulp cells (DPCs) were positive for mesenchymal stem cell markers, including stage-specific embryonic antigen 4 (SSEA-4), CD146, CD29, CD44, and CD90, but were negative for hematopoietic stem cell markers CD34 and CD45. Click here for file

Additional file 2: Figure S2 Optimization of the experimental protocol. Analysis of cell viability of dental pulp cells (DPCs) was measured by MTS (3-(4,5-dimethylthiazol-2-yl)-5-(3-carboxymethoxyphenyl)-2-(4-sulfophenyl)-2H-tetrazolium) assay. **(A)** No significant difference in cell viability was observed when DPCs were stimulated with increasing doses of tumor necrosis factor-alpha (TNF-α) for 3 days. **(B)** A time-dependent analysis of cell viability after DPCs were incubated with TNF-α (10 ng/mL) for 2, 4, or 6 days. A significant decrease in cell viability was observed only when the incubation period extended for 6 consecutive days. ***P <0.001 (one way analysis of variance/Tukey) compared with day-0 group. **(C)** Left panels show the differentiation capacity of DPCs toward odontogenic and adipogenic lineage. Right panels show that a long-term TNF-α treatment (odontogenic differentiation = 21 days, adipogenic differentiation = 30 days) suppresses DPC differentiation. **(D)** TNF-α-induced increase in stage-specific embryonic antigen 4 (SSEA-4) levels was similar with different concentrations of the cytokine. **(E,F)** TNF-α stimulation for 4 days did not increase either the number of SSEA-4^+^ cells or mRNA levels of octamer-binding transcription factor 4 (OCT-4). All experiments were performed with at least triplicate samples. Click here for file
