# Supplementary figures and images for: A short-term treatment with tumor necrosis factor-alpha enhances stem cell phenotype of human dental pulp cells
Source: Stem Cell Res Ther. 2014 Feb 28;5(1):31. doi: 10.1186/scrt420 (PMC4055131; doi:10.1186/scrt420)

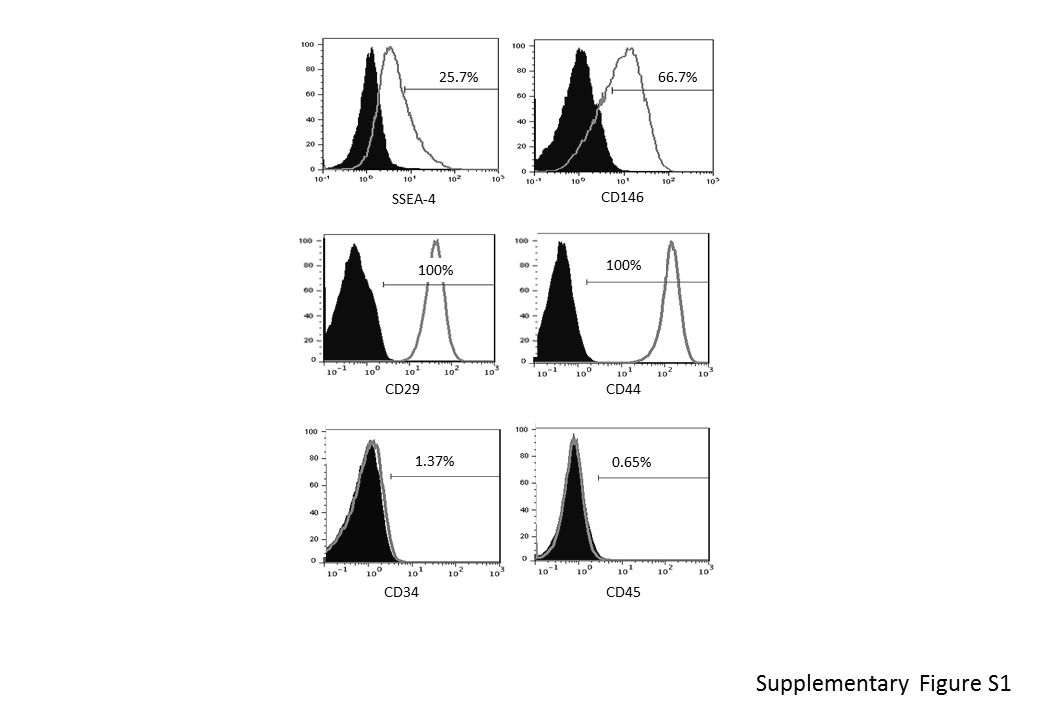

Supplement: Additional file 1: Figure S1 — Analysis of cell surface markers by flow cytometry. Isolated dental pulp cells (DPCs) were positive for mesenchymal stem cell markers, including stage-specific embryonic antigen 4 (SSEA-4), CD146, CD29, CD44, and CD90, but were negative for hematopoietic stem cell markers CD34 and CD45. [file scrt420-S1.tiff]

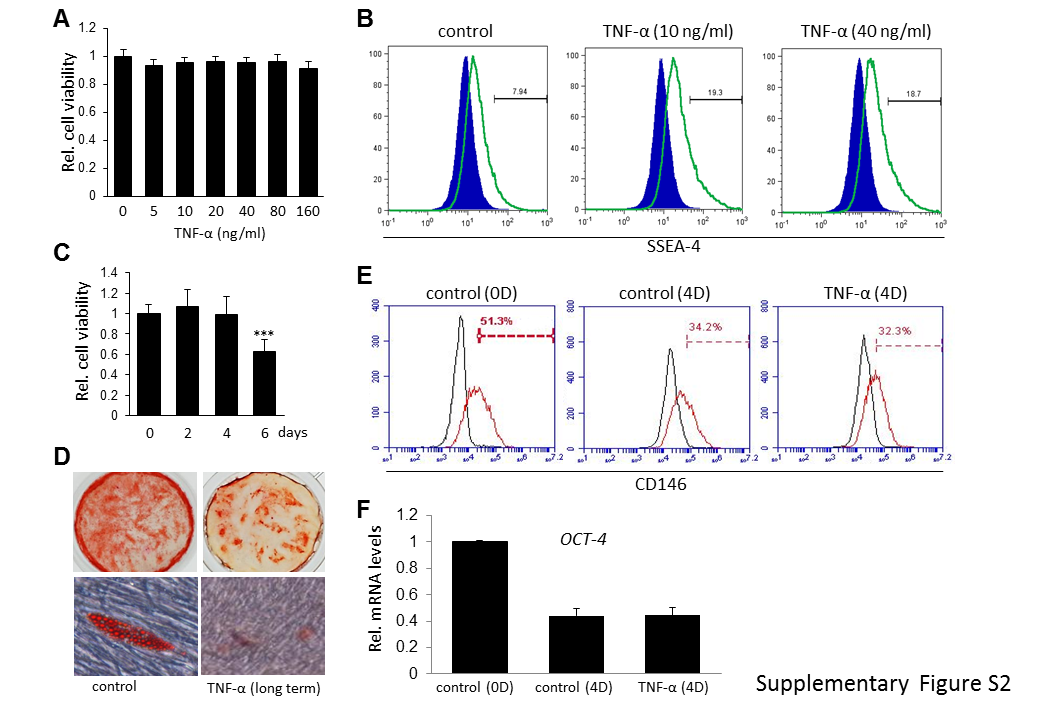

Supplement: Additional file 2: Figure S2 — Optimization of the experimental protocol. Analysis of cell viability of dental pulp cells (DPCs) was measured by MTS (3-(4,5-dimethylthiazol-2-yl)-5-(3-carboxymethoxyphenyl)-2-(4-sulfophenyl)-2H-tetrazolium) assay. (A) No significant difference in cell viability was observed when DPCs were stimulated with increasing doses of tumor necrosis factor-alpha (TNF-α) for 3 days. (B) A time-dependent analysis of cell viability after DPCs were incubated with TNF-α (10 ng/mL) for 2, 4, or 6 days. A significant decrease in cell viability was observed only when the incubation period extended for 6 consecutive days. ***P <0.001 (one way analysis of variance/Tukey) compared with day-0 group. (C) Left panels show the differentiation capacity of DPCs toward odontogenic and adipogenic lineage. Right panels show that a long-term TNF-α treatment (odontogenic differentiation = 21 days, adipogenic differentiation = 30 days) suppresses DPC differentiation. (D) TNF-α-induced increase in stage-specific embryonic antigen 4 (SSEA-4) levels was similar with different concentrations of the cytokine. (E,F) TNF-α stimulation for 4 days did not increase either the number of SSEA-4+ cells or mRNA levels of octamer-binding transcription factor 4 (OCT-4). All experiments were performed with at least triplicate samples. [file scrt420-S2.tiff]
